# The need to monitor actions on the social determinants of health

**DOI:** 10.2471/BLT.16.184622

**Published:** 2017-09-12

**Authors:** Frank Pega, Nicole B Valentine, Kumanan Rasanathan, Ahmad Reza Hosseinpoor, Tone P Torgersen, Veerabhadran Ramanathan, Tipicha Posayanonda, Nathalie Röbbel, Yassine Kalboussi, David H Rehkopf, Carlos Dora, Eugenio R Villar Montesinos, Maria P Neira

**Affiliations:** a Department of Public Health, Environmental and Social Determinants of Health, World Health Organization, Avenue Appia 20, 1211 Geneva 27, Switzerland.; b Health Section, United Nations Children’s Fund (UNICEF), New York, NY 10017, United States of America (USA).; c Department of Information, Evidence and Research, World Health Organization, Geneva, Switzerland.; d Department of Social Determinants of Health, Norwegian Directorate of Health, Oslo, Norway.; e Scripps Institution of Oceanography, University of California San Diego, La Jolla, USA.; f National Health Commission Office, Nonthaburi, Thailand.; g Department of Medicine, Stanford University, Stanford, USA.

Intersectoral actions, defined as the alignment of strategies and resources between actors from two or more policy sectors to achieve complementary objectives,^1^ are central to the health-related sustainable development goals (SDGs).^2^ The World Health Organization’s (WHO) Commission on Social Determinants of Health recommended a subset of intersectoral actions to improve health equity in 2008.^3^ Intersectoral actions address the social, commercial, cultural, economic, environmental and political determinants of health. Without intersectoral actions, the health sector will probably not achieve SDG 3, that is, ensuring healthy lives and promoting well-being for all at all ages.^4^

National governments have committed to and implemented several of these intersectoral actions through multisectoral development and health policy frameworks, including the *2030 agenda for sustainable development*,^4^ the Rio Political Declaration on Social Determinants of Health,^5^ the New Urban Agenda^6^ and the Marrakech Ministerial Declaration on Health, Environment and Climate Change.^7^

We argue for monitoring intersectoral actions because such assessment draws attention to those government interventions that improve living conditions, but are outside the immediate control of the health sector. These interventions often have established co-benefits across multiple policy sectors (for instance, emission-free public transport systems improve air quality, transport and health). Action monitoring can also strengthen coherence and efficiency across sectors. The SDGs’ extensive multisectoral indicator framework^8^ offers health policy-makers the opportunity to link action monitoring to the SDGs, as national governments begin their SDG implementation.^4^

In particular, actions taken in the context of policy frameworks that address the social determinants of health, such as those in the five action areas of the Rio Political Declaration,^5^ need to be monitored. Therefore, we define and categorize indicators for intersectoral actions on social determinants of health that improve health equity. If these indicators are drawn from the SDG indicator system,^8^ they will enable policy-makers to link intersectoral actions to sustainable development.^9^

For social determinants of health, we use WHO commission’s definitions,^3^ which refer to the wider set of social, commercial, cultural, economic, environmental and political determinants that drive patterns of health inequalities. These determinants are the daily conditions in which people grow, live, work and age; they are the forces and systems shaping living conditions. Determinants include population exposure to the physical environment; occupational hazards, housing, chemicals, air and water quality, sanitation and hygiene, and climate change. The determinants converge and accumulate over time to shape the health of population groups according to their social status. This is defined by, for example, education, ethnicity including indigeneity and migrant status, gender, gender identity, income, occupation and sexual orientation. Hence, changes in health equity that result from specific interventions or policy frameworks aimed to improve social determinants of health may take time to show.

Using the commission’s evidence-based recommendations for intersectoral action,^3^ we offer a classification of three groups of intersectoral interventions that focus on the determinants and are relevant to the SDGs’ equity and sustainable development foci.^4^ The first group includes governance interventions, defined as political and decision-making structures and processes that improve health equity, such as whole-of-government or multisectoral committees, funds or plans, or human rights legislation. The second group consists of socioeconomic interventions, defined as those policies and programmes that allocate social and or financial resources to improve health equity. Such interventions could improve early child development, education, living wage, pay equity and social protection. The third group includes environmental interventions, defined as policies or programmes for the built or natural environment that improve health equity. Examples of such interventions are slum upgrading, air and drinking water quality improvement, sanitation and hygiene improvement and climate change mitigations and adaptations. Of the commission’s 39 intersectoral action recommendations,^3^ 17 are for governance interventions, 16 for socioeconomic interventions and 6 for environmental interventions (Box 1).

Box 1 **Recommendations from the Commission on Social Determinants of Health for intersectoral action on the social determinants of health**

**Intersectoral governance interventions**

Local government and civil society, backed by national government, establish local participatory governance mechanisms that enable communities and local government to partner in building healthier and safer cities.

Parliament and equivalent oversight bodies adopt a goal of improving health equity through action on the social determinants of health as a measure of government performance.

National government establish a whole-of-government mechanism that is accountable to parliament, chaired at the highest political level possible.

The monitoring of social determinants and health equity indicators be institutionalized and health equity impact assessment of all government policies, including finance, be used.

National and local governments and civil society establish a cross-government mechanism to allocate budget to action on social determinants of health.

Public resources be equitably allocated and monitored between regions and social groups, for example, using an equity gauge.

Government policy-setting bodies ensure and strengthen representation of public health in domestic and international economic policy negotiations.

Governments create and enforce legislation that promotes gender equity and makes discrimination on the basis of sex illegal.

Governments set up within the central administration and provide adequate and long-term funding for a gender equity unit that is mandated to analyse and to act on the gender equity implications of policies, programmes, and institutional arrangements.

National government strengthens the political and legal systems to ensure they promote the equal inclusion of all.

National government acknowledges, legitimizes, and supports marginalized groups, in particular Indigenous Peoples in policy, legislation, and programmes that empower people to represent their needs, claims, and right.

National- and local-level government ensure the fair representation of all groups and communities in decision-making that affects health, and in subsequent programme and service delivery and evaluation.

Support for civil society to develop, strengthen, and implement health equity-oriented initiatives.

Governments ensure that all children are registered at birth without financial cost to the household.

National governments establish a national health equity surveillance system, with routine collection of data on social determinants of health and health inequity.

Governments build capacity for health equity impact assessment among policy-makers and planners across government departments.

Governments include the economic contribution of household work, care work, and voluntary work in national accounts and strengthen the inclusion of informal work.

**Intersectoral socioeconomic interventions**

Build comprehensive package of quality early child development programmes and services for children, mothers, and other caregivers, regardless of ability to pay.

Provide quality education that pays attention to children’s physical, social/emotional, and language/cognitive development, starting in pre-primary school.

Provide quality compulsory primary and secondary education for all boys and girls, regardless of ability to pay, identify and address the barriers to girls and boys enrolling and staying in school.

Develop and implement economic and social policies that provide secure work and a living wage that takes into account the real and current cost of living for health.

Build universal social protection systems and increase their generosity towards a level that is sufficient for healthy living.

Use targeting only as back up for those who slip through the net of universal systems.

Ensure that social protection systems extend to include those who are in precarious work, including informal work and household or care work.

Invest in expanding girls’ and women’s capabilities through investment in formal and vocational education and training.

Support women in their economic roles by guaranteeing pay-equity by law, ensuring equal opportunity for employment at all levels, and by setting up family-friendly policies that ensure that women and men can take on care responsibilities in an equal manner.

Research funding bodies create a dedicated budget for generation and global sharing of evidence on social determinants of health and health equity, including health equity intervention research.

Educational institutions and relevant ministries make the social determinants of health a standard and compulsory part of training of medical and health professionals.

Educational institutions and relevant ministries act to increase understanding of the social determinants of health among non-medical professionals and the general public.

Reduce insecurity among people in precarious work arrangements including informal work, temporary work, and part-time work through policy and legislation to ensure that wages are based on the real cost of living, social security, and support for parents.

Develop and implement economic and social policies that provide secure work and a living wage that takes into account the real and current cost of living for health.

Build and strengthen national capacity for progressive taxation.

New national and global public finance mechanisms be developed, including special health taxes and global tax options.

**Intersectoral environmental interventions**

Manage urban development to ensure greater availability of affordable quality housing; invest in urban slum upgrading including, as a priority, provision of water and sanitation, electricity, and paved streets for all.

Plan and design urban areas to promote physical activity through investment in active transport; encourage healthy eating through retail planning to manage the availability of and access to food; and reduce violence and crime through good environmental design and regulatory controls.

Develop and implement policies and programmes that focus on: issues of rural land tenure and rights; year-round rural job opportunities; agricultural development and fairness in international trade arrangements; rural infrastructure including health, education, roads, and services; and policies that protect the health of rural-to-urban migrants.

Occupational health and safety policy and programmes be applied to all workers – formal and informal – and that the range be expanded to include work-related stressors and behaviours as well as exposure to material hazards.

Strengthen public sector leadership in the provision of essential health-related goods/services and control of health-damaging commodities.

Public capacity be strengthened to implement regulatory mechanisms to promote and enforce fair employment and decent work standards for all workers.

Effective action monitoring requires valid, sensitive and reliable indicators drawn from a solid evidence base on intervention effectiveness. Theoretical evidence suggests that interventions focussed on social determinants of health could be used as action indicators, since they are theorized to improve these determinants, health service use, health outcomes and health equity.^3^ Social epidemiological methods, which often rely on natural experiments to support causal inference, have been developed to evaluate the impact of interventions on the social determinants of health.^10^ Empirical evidence, including systematic reviews, supports the use of specific socioeconomic interventions as action indicators on the social determinants of health. Evidence for social protection interventions is particularly developed. In contrast, more research on the effectiveness of governance and environmental interventions is needed. 

WHO’s standard classification^11^ of health systems performance indicators has four groups according to function in the production cycle: (i) inputs – quantity or quality of resources provided by an intervention and the processes for combining them; (ii) outputs – quantity or quality of the results achieved by the input; (iii) policy or programme outcomes – the intervention’s population coverage; and (iv) impacts on end goals – well-being and health outcomes influenced by the intervention, such as improved social determinants of health and health equity. Action indicators should be constituted from input, output and outcomes indicators, but not from impact indicators.

**Fig. 1:**
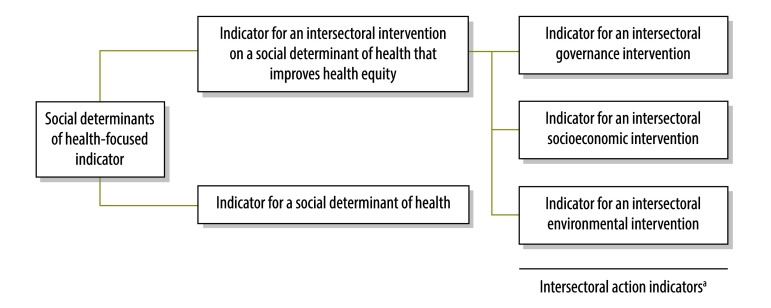
Proposed classification of social determinants of health-focused indicators

^a^ Action indicators focus on interventions on social determinant of health that improve health equity.

Fig. 1 shows our proposed classification of indicators on social determinants of health. We suggest distinguishing between indicators for an intersectoral action that improves health equity and indicators for a social determinant of health per se. We broadly define a social determinant of health action indicator as one for an intersectoral governance, socioeconomic or environmental intervention that improves health equity (Table 1).

**Table 1:** Proposed social determinants of health action indicator subgroups, definitions and examples of related SDG indicators

Indicator subgroup	Definition	Example from the SDGs monitoring system^8^
Indicator for an intersectoral governance intervention	Indicator for an intersectoral political or decision-making structure or process that improves health equity	Indicator 6.b.1 Proportion of local administrative units with established and operational policies and procedures for participation of local communities in water and sanitation management
Indicator for an intersectoral socioeconomic intervention	Indicator for an intersectoral policy or programme allocating social, financial or economic resources that improves health equity	Indicator 1.3.1 Proportion of the population covered by social protection floors / systems disaggregated by sex and distinguishing children, unemployed, old age, people with disabilities, pregnant women / new-borns, work injury victims, poor and vulnerable
Indicator for an intersectoral environmental intervention	Indicator for an intersectoral policy or programme for the built or natural environment that improves health equity	Indicator 11.6.1 Proportion of urban solid waste regularly collected and with adequate final discharge with regards to the total waste generated by the city

SDGs: sustainable development goals.

We suggest that action monitoring be integrated in broader SDG monitoring. This would avoid placing the burden of reporting on the health sector and avoid additional reporting burden on countries (Table 1).

There are established systems for monitoring health inequalities, such as WHO’s Health Equity Monitor,^12^ and for monitoring the social determinants of health per se. However, the effects of an intersectoral intervention may take a long time to show in such monitoring, and observed changes in health inequalities or in a social determinant of health cannot necessarily be attributed to the intervention. Therefore, systems are needed to monitor intersectoral actions on social determinants of health. WHO is already developing such international monitoring systems,^13^^,^^14^ and Canada has started reporting on national and subnational actions taken to implement the Rio Political Declaration.^15^ Action indicators should be included in established systems for monitoring health inequalities, social determinants of health and the health-in-all-policies approach. Civil society and multistakeholder initiatives, such as Countdown to 2030,^16^ may also contribute to action monitoring.

Comprehensive action monitoring on these determinants requires a sensible selection of indicators from all three intervention groups. Indicators could be drawn from non-health sectors and or from multisectoral indicator sets and databases. Health ministries in Ecuador, New Zealand and Norway have gathered indicators in this way to monitor social determinants of health. The SDG indicators^8^ – multisectoral and common to all countries – should also be a good source of action indicators.
